# Overexpression of Isoforms of Nitric Oxide Synthase 1 Adaptor Protein, Encoded by a Risk Gene for Schizophrenia, Alters Actin Dynamics and Synaptic Function

**DOI:** 10.3389/fncel.2016.00006

**Published:** 2016-02-02

**Authors:** Kristina Hernandez, Przemyslaw Swiatkowski, Mihir V. Patel, Chen Liang, Natasha R. Dudzinski, Linda M. Brzustowicz, Bonnie L. Firestein

**Affiliations:** ^1^Department of Cell Biology and Neuroscience, Human Genetics Institute of New Jersey, Rutgers—The State University of New JerseyPiscataway, NJ, USA; ^2^Department of Cell Biology and Neuroscience, Rutgers—The State University of New JerseyPiscataway, NJ, USA; ^3^Department of Genetics, Human Genetics Institute of New Jersey, Rutgers—The State University of New JerseyPiscataway, NJ, USA

**Keywords:** NOS1AP, actin, dendritic spines, cortical neurons, schizophrenia, synaptic strength

## Abstract

Proper communication between neurons depends upon appropriate patterning of dendrites and correct distribution and structure of spines. Schizophrenia is a neuropsychiatric disorder characterized by alterations in dendrite branching and spine density. *Nitric oxide synthase 1 adaptor protein (NOS1AP)*, a risk gene for schizophrenia, encodes proteins that are upregulated in the dorsolateral prefrontal cortex (DLPFC) of individuals with schizophrenia. To elucidate the effects of NOS1AP overexpression observed in individuals with schizophrenia, we investigated changes in actin dynamics and spine development when a long (NOS1AP-L) or short (NOS1AP-S) isoform of NOS1AP is overexpressed. Increased NOS1AP-L protein promotes the formation of immature spines when overexpressed in rat cortical neurons from day *in vitro* (DIV) 14 to DIV 17 and reduces the amplitude of miniature excitatory postsynaptic currents (mEPSCs). In contrast, increased NOS1AP-S protein increases the rate of actin polymerization and the number of immature and mature spines, which may be attributed to a decrease in total Rac1 expression and a reduction in the levels of active cofilin. The increase in the number of mature spines by overexpression of NOS1AP-S is accompanied by an increase in the frequency of mEPSCs. Our findings show that overexpression of NOS1AP-L or NOS1AP-S alters the actin cytoskeleton and synaptic function. However, the mechanisms by which these isoforms induce these changes are distinct. These results are important for understanding how increased expression of NOS1AP isoforms can influence spine development and synaptic function.

## Introduction

**Figure 1:**
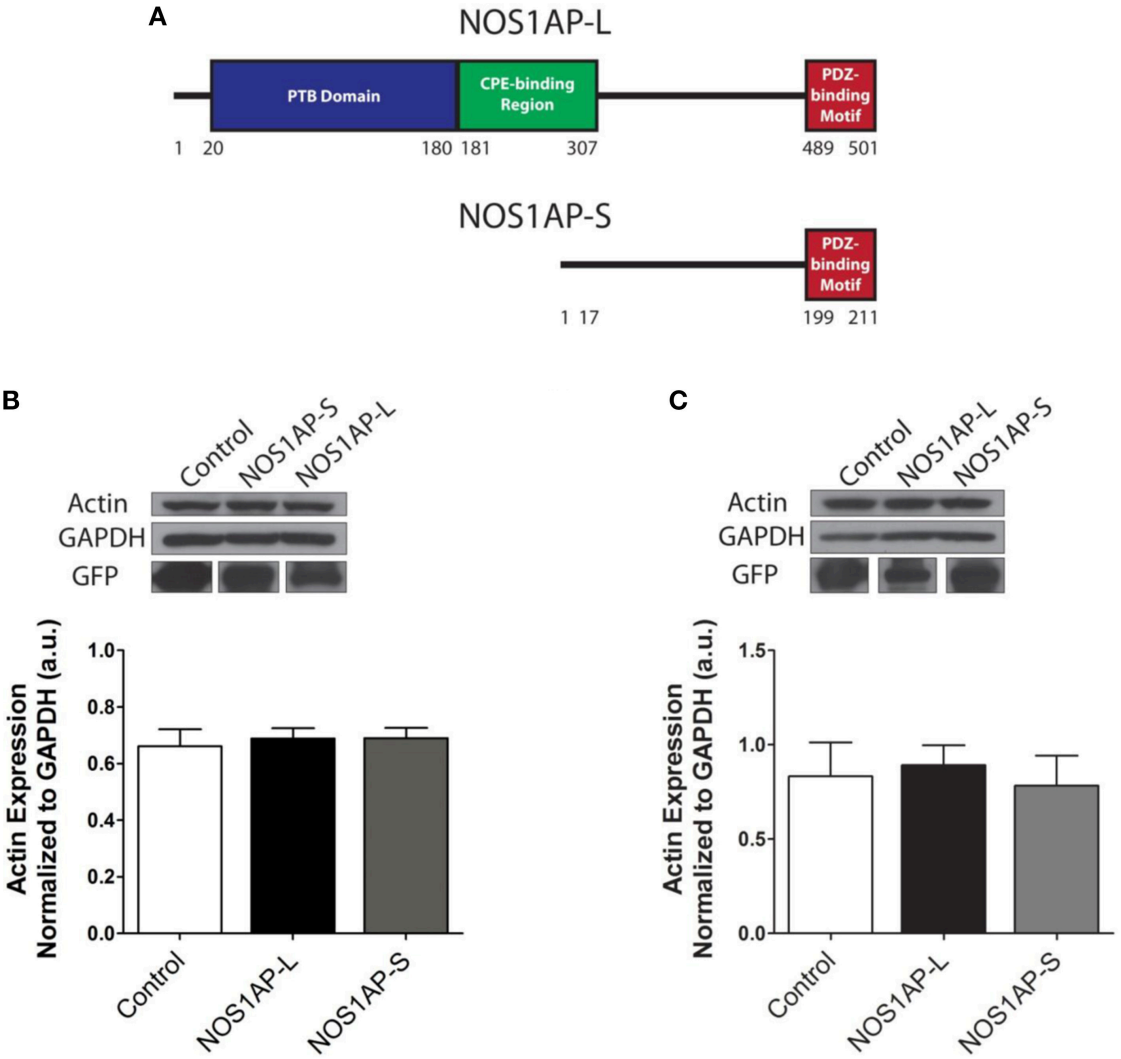
**Expression of NOS1AP-L or NOS1AP-S in COS-7 cells decreases F-actin. (A)** Schematic of NOS1AP-Long and NOS1AP-Short protein domains. The long isoform of NOS1AP produces a protein with a PTB domain, CPE-binding region, and a PDZ-binding motif (NOS1AP-L). The short isoform of NOS1AP produces a protein with only the PDZ-binding motif (NOS1AP-S). The first 17 amino acids of NOS1AP-S are not present in NOS1AP-L. **(B)** *Upper*, extracts from cultures of transfected COS-7 cells expressing GFP (control), NOS1AP-L, or NOS1AP-S were resolved by SDS-PAGE and analyzed by Western blotting using antibodies that recognize actin or GAPDH. Representative blot is shown. *Lower*, densitometry analysis of multiple blots represented in *upper*. Error bars indicate ± s.e.m. *n* = 6 for all conditions. a.u., arbitrary units. **(C)** *Upper*, extracts from cultures of transfected HEK293T cells expressing GFP (control), NOS1AP-L, or NOS1AP-S were resolved by SDS-PAGE and analyzed by Western blotting using antibodies that recognize actin or GAPDH. Representative blot is shown. *Lower*, densitometry analysis of multiple blots represented in *upper*. Error bars indicate ± s.e.m. *n* = 5 for all conditions. a.u., arbitrary units.

Nitric Oxide Synthase 1 Adaptor Protein (NOS1AP) was originally identified as a negative regulator of the interaction between the enzyme neuronal nitric oxide synthase (NOS1; nNOS) and PSD-95 (Jaffrey et al., 1998). Multiple isoforms of NOS1AP exist, including long (NOS1AP-L) and short (NOS1AP-S) isoforms (Figures 1A,B). NOS1AP-L consists of 501 amino acids and contains an amino-terminal phosphotyrosine-binding (PTB) domain and a carboxyl-terminal PDZ-binding motif. NOS1AP-S consists of 211 amino acids and also contains the PDZ-binding motif at its carboxyl-terminus. The PTB domain of NOS1AP-L binds to Dexras1, synapsin, and Scribble (Fang et al., 2000; Jaffrey et al., 2002; Richier et al., 2010) and is responsible for the disruption of neuronal migration by NOS1AP-L during cortical development (Carrel et al., 2015). The PDZ-binding motif is important for stabilization of the binding of NOS1AP to neuronal nitric oxide synthase 1 (NOS1; Jaffrey et al., 1998; Li et al., 2015), influencing nNOS localization, and therefore, mediating nitric oxide (NO) signaling. Overexpression of NOS1AP-L negatively regulates dendrite branching during multiple developmental stages in rat hippocampal neurons, while overexpression of NOS1AP-S only affects early dendrite development (Carrel et al., 2009). In addition, NOS1AP-L associates with the tumor suppressor protein, Scribble, to regulate spine development (Richier et al., 2010).

Dynamic reorganization of the actin cytoskeleton of neurons is essential for numerous developmental processes that are regulated by NOS1AP isoforms, such as dendritic growth and spine development (Georges et al., 2008; Hotulainen and Hoogenraad, 2010). Furthermore, changes in the structure or number of spines have implications for altered synaptic plasticity and function (Kasai et al., 2010). The Rho family of GTPases is composed of key intracellular regulators of spine development (Nakayama et al., 2000; Tashiro et al., 2000), such as Rac1, that act by influencing the actin cytoskeleton (Hall, 1994). A common signaling pathway among several small GTPases involves the regulation of cofilin activity. Cofilin is a member of the actin depolymerizing factor (ADF)/cofilin family of proteins that enhances the rate of actin filament turnover, both *in vivo* and *in vitro*, by severing and depolymerizing actin filaments (Carlier et al., 1997; Lappalainen and Drubin, 1997). The activity of cofilin is regulated by the phosphorylation of its Ser-3 residue, resulting in its inactivation (Moriyama et al., 1996). The inactivation of cofilin promotes increased stabilization of actin filaments and actin polymerization (Tybulewicz and Henderson, 2009). In neurons, cofilin has been shown to be important for spine remodeling and synaptic plasticity. Activity-dependent spine growth is coupled to cofilin phosphorylation, which induces actin polymerization (Chen et al., 2007; Calabrese et al., 2014).

NOS1AP is a protein encoded by a schizophrenia susceptibility gene (Brzustowicz et al., 2000; Zheng et al., 2005; Miranda et al., 2006; Kremeyer et al., 2008; Wratten et al., 2009). We have previously shown that the expression of NOS1AP-L and NOS1AP-S is upregulated in postmortem tissue from the dorsolateral prefrontal cortex (DLPFC) of individuals with schizophrenia (Hadzimichalis et al., 2010). Since schizophrenia is believed to be a neurodevelopmental disorder (Benes, 1991; Murray et al., 1991; Bunney et al., 1995; Brent et al., 2014), in the present study, we investigate the effects of overexpression of these isoforms on actin dynamics, dendritic spine number and morphology, and resulting electrophysiology. We report that both NOS1AP-L and NOS1AP-S associate with filamentous actin (F-actin) and that increased protein levels of NOS1AP-L or NOS1AP-S result in reorganization of the actin cytoskeleton. In addition, overexpression of either NOS1AP-L or NOS1AP-S differentially perturbs spinogenesis in rat cortical neurons, resulting in altered synaptic function. Interestingly, we find that the mechanisms by which NOS1AP-L and NOS1AP-S may influence these processes differ at the molecular level. Specifically, the overexpression of NOS1AP-S results in the down regulation of Rac1 protein expression and reduced levels of the active, non-phosphorylated form of cofilin.

## Materials and methods

### Antibodies

Rabbit polyclonal NOS1AP (sc-9138) and goat polyclonal DNase I antibodies were from Santa Cruz Biotechnology (Santa Cruz, CA). Mouse monoclonal GAPDH antibody was from Millipore (Billerica, MA), mouse monoclonal anti-alpha-actinin-4 from Abcam (Cambridge, MA), and mouse monoclonal anti-actin from Sigma-Aldrich (St. Louis, MO). Chicken and goat polyclonal green fluorescent protein (GFP) antibodies were from Rockland Immunochemicals (Limerick, PA). Rabbit polyclonal actin and mouse monoclonal Rac1 antibodies were from Cytoskeleton, Inc (Denver, CO). Alexa Fluor® 647 phalloidin, chicken secondary antibody conjugated to Alexa-Fluor® 488, and mouse monoclonal anti-nNOS were from Life Technologies (Grand Island, NY). Mouse monoclonal cofilin antibody was from BD Biosciences (San Jose, CA) and rabbit monoclonal Phospho-cofilin (Ser3) antibody was from Cell Signaling Technology (Danvers, MA).

### DNA constructs

pCAG-GFP was obtained by subcloning EGFP from pEGFP-C1 (Clontech; Mountain View, CA) into a vector with CMV–actin–β-globin promoter (pCAG). cDNAs encoding long and short isoforms of human NOS1AP (NOS1AP-L and NOS1AP-S), NOS1AP-S-1-197 (NOS1AP-S-ΔPDZ), NOS1AP-L-214-end (NOS1AP-L-ΔPTB), NOS1AP-L-1-487 (NOS1AP-L-ΔPDZ), and NOS1AP-L-181-307 (NOS1AP-M) were sub cloned into pCAG-GFP as described previously (Carrel et al., 2009).

### Transfection of COS-7 cells and immunocytochemistry

COS-7 cells were plated onto 0.1 mg/ml poly-d-lysine hydrobromide (Sigma-Aldrich)-coated coverslips at 10,550 cells/cm^2^ and transfected with pCAG-GFP, pCAG-NOS1AP-L, pCAG-NOS1AP-S, pCAG-NOS1AP-S-ΔPDZ, pCAG-NOS1AP-L-ΔPTB, pCAG-NOS1AP-L-ΔPDZ, or pCAG-NOS1AP-M using Lipofectamine 2000 (Life Technologies) following the manufacturer's protocol. Forty-eight hours after transfection, cells were fixed with 4% paraformaldehyde in phosphate-buffered saline for 15 min and immunostained for GFP using chicken anti-GFP and Alexa-Fluor® 488 anti-chicken and for filamentous actin (F-actin) using Alexa-Fluor® 647-Phalloidin, followed by nuclear staining with Hoechst dye. Coverslips were mounted onto glass slides using Fluoromount G (Southern Biotechnology; Birmingham, AL).

### Measurement of F-actin and quantitation of protrusions in COS-7 cells

Cells were imaged at 600x with a fixed exposure time among experimental conditions using an Olympus Optical (Tokyo, Japan) IX50 microscope with a Cooke Sensicam CCD cooled camera, fluorescence imaging system, and ImagePro software (MediaCybernetics; Silver Spring, MD). For F-actin content analysis, the outer boundaries of individual transfected cells were traced using ImageJ (NIH; Bethesda, MD) with the experimenter blinded to the condition to quantify the mean fluorescence intensity of Alexa Fluor® 647-phalloidin staining within each cell. Mean fluorescence intensity is defined as the sum of the gray values of all the pixels in the selection divided by the total number of pixels. For each image, an average of the mean fluorescence intensity of the background was calculated and subtracted from the mean fluorescence intensity of the transfected cell. To quantify the protrusion index, the outer boundary of a transfected cell (including all branched and unbranched protrusions; area A) was traced and the periphery of that cell excluding all protrusions was traced (area B). The two areas were calculated, and the latter area was subtracted from the former to give the area occupied by the protrusions. This area was divided by the length of periphery of B to give the protrusion index (Lin et al., 2010).

### Western blotting of COS-7 cell lysates

COS-7 cells were cultured in 60 mm dishes and transfected at 30–50% confluency with pCAG-GFP, pCAG-GFP-NOS1AP-L, or pCAG-GFP-NOS1AP-S using Lipofectamine 2000 following the manufacturer's protocol. Cells were collected 2 days after transfection and lysed, and expression of actin, GFP, and GAPDH was detected by immunoblotting after resolving proteins using SDS-PAGE. After electrophoresis, proteins were transferred to PVDF membranes (Immobilon-P; Millipore). After blocking with 2% bovine serum albumin (BSA) in Tris-buffered saline (500 mM Tris, pH 7.4, 60 mM KCl, 2.8 M NaCl) with 1% Tween-20 (TBST), membranes were incubated with primary antibodies overnight at 4°C: 1:1000 for mouse anti-actin, 1:1000 for mouse anti-GAPDH, or 1:500 for goat anti-GFP. After washing, horseradish peroxidase-linked secondary antibody was applied at 1:5000 for 1 h at RT. Immunoreactive bands were visualized using HyGlo quick spray (Denville Scientific; South Plainfield NJ) and quantified using Image Pro software (Media Cybernetics).

### Western blotting of HEK293T cell lysates

HEK293T cells were transfected (30–50% confluency) with pCAG-GFP, pCAG-GFP-NOS1AP-L, or pCAG-GFP-NOS1AP-S using the calcium phosphate method (Kwon and Firestein, 2013), incubated overnight, and incubated in serum-free medium for an additional 24 h. Medium was changed to serum-containing medium for 10 min before scrape-harvesting protein. Cells were harvested in lysis buffer (50 mM Tris pH 7.5, 10 mM MgCl2, 0.5 M NaCl, and 2% Igepal) supplemented with protease inhibitors (62 μg/ml Leupeptin, 62 Ég/ml Pepstatin A, 14 mg/ml Benzamidine, and 12 mg/ml tosyl arginine methyl ester) and 1 mM sodium orthovanadate, pH 10. Western blotting was performed for total Rac1 using mouse anti-Rac1, cofilin using mouse anti-cofilin, phosphorylated-cofilin using rabbit anti-Phospho-cofilin (Ser3), and GAPDH using mouse anti-GAPDH. Immunoreactive bands were quantified using Image Pro software.

### F-actin immunoprecipitation

Adult rat brain tissue was homogenized and lysed in F-actin stabilization/lysis buffer (50 mM PIPES pH 6.9, 50 mM KCl, 5 mM MgCl^2^, 5 mM EGTA, 5% (v/v) glycerol, 0.1% nonidet P40, 0.1% Triton X-100, 0.1% Tween 20, 0.1% 2-mercaptoethanol, and 1 mM ATP) supplemented with 1 Complete, Mini, EDTA-free protease inhibitor tablet (Roche Diagnostics, Mannheim, Germany). Extracted proteins were diluted two-fold, and 500 μl extract was incubated without (negative control) or with 5 μg biotin labeled-phalloidin (Sigma-Aldrich) followed by precipitation of captured complexes with 20 μl streptavidin-linked magnetic beads (Dynabeads® M-280 Streptavidin, Life Technologies). Beads were washed three times with PBS, pH 7.4, with samples used for biochemical analysis after third wash. Precipitated fractions were subjected to Western blotting to detect NOS1AP isoforms, alpha-actinin-4, Dnase I, and actin.

### Rat brain co-immunoprecipitation

Rat brain was homogenized in TEEN [25 mM Tris-HCl, pH 7.4, 1 mM EDTA, 1 mM EGTA, 100 mM NaCl), 1 mM phenylmethylsulfonylfluoride (PMSF), 1 mM sodium orthovanadate, pH 10, and one protease inhibitor tablet (Roche Diagnostics)]. Triton X-100 was added to a final concentration of 1%, and proteins were extracted at 4°C for 1 h. Detergent-insoluble material was pelleted by centrifugation at 12,000 × g at 4°C for 15 min. Lysate was pre-cleared with protein G agarose 50% slurry (GE Healthcare, Piscataway, NJ) for one and a half hour and then subjected to immunoprecipitation with monoclonal nNOS antibody or mouse IgG at 4°C for 2 h. Immunoprecipitates were washed three times with TEEN containing 0.1% Triton X-100, and bound proteins were eluted with protein loading buffer. Eluates were subjected to Western blotting to detect nNOS and NOS1AP isoforms.

### *In vitro* pyrene-actin polymerization assays

The rate of non-muscle actin polymerization in the presence of lysates from cultures overexpressing GFP, GFP-NOS1AP-L, or GFP-NOS1AP-S was monitored according to the methods outlined in the Actin Polymerization Biochem Kit (Cytoskeleton, Inc.). HEK293T cells were cultured in 10 cm dishes and transfected at 30–50% confluency with NOS1AP constructs using calcium phosphate method. Forty-eight hours later, total protein was extracted in Buffer A [20 mM Tris-HCl, pH 7.5, 20 mM NaCl, 1% Triton X-100, 1 mM phenylmethylsulfonyl fluoride (PMSF)]. Protein lysates were diluted to 1.5 mg/ml with Buffer A lacking Triton X-100 for final 0.1% [Triton X-100]. Pyrene-labeled rabbit muscle actin and human non-muscle actin (Cytoskeleton, Inc.) were mixed 1:10 to monitor non-muscle actin polymerization. The pyrene-muscle actin and unlabeled non-muscle actin mixture was diluted to 0.45 mg/ml in G-buffer. Pyrene muscle actin will not polymerize efficiently on its own at the concentration used in this assay, so the reaction is dependent on non-muscle actin polymerization for F-actin formation. *In vitro* polymerization assays (200 μl) were performed in black with clear bottom 96-well plates (Corning; Corning, NY). Duplicate or triplicate wells were assayed for G-buffer; pyrene-actin, lysis buffer (20 mM Tris-HCl, pH 7.5, 20 mM NaCl, 0.1% Triton X-100, 1 mM PMSF); pyrene-actin, GFP; pyrene-actin, NOS1AP-L; and pyrene-actin, NOS1AP-S. Polymerization reactions were started 30 s prior to measurement by addition of 20 μl 10X actin polymerization buffer. Increase in fluorescence following polymerization was measured with CytoFluor Series 4000 fluorescence plate reader (Applied Biosystems, Life Technologies): excitation, 360 ± 40 nm, emission, 460 ± 40 nm every 30 s. To quantify changes in polymerization rate, linear regression was performed using GraphPad, Prism (San Diego, CA) to calculate the V_max_ for the growth phase of polymerization.

### Primary cortical neuron culture and spine analysis

Neuronal cultures were plated from cortices of rat embryos at 18 days gestation on glass coverslips (12 mm diameter; 53,000 cells/cm^2^), as previously reported (Carrel et al., 2009). At day *in vitro* (DIV) 14, cultures were transfected with indicated constructs using calcium phosphate method. Neurons were fixed at DIV 17 and immunostained for GFP. Images of dendritic segments were taken with a high numerical aperture objective lens (40x C. Apochromat, N.A. 1.2) on a laser scanning confocal microscope, LSM510 META (Carl Zeiss Microscopy; Thornwood, NY). X-,Y-, and Z-resolution was set as 0.1, 0.1, and 0.3 μm, respectively, to define dendritic spines. Additionally, images of dendritic segments of neurons transfected with NOS1AP-S or NOS1AP-S-ΔPDZ were taken using a 60x plan apochromatic oil-immersion objective (NA 1.4) using a Yokogawa CSU-10 spinning disk confocal head attached to an inverted fluorescence microscope (Olympus IX50). X-, Y-, and Z-resolution were set as 0.067, 0.067, and 0.1 μm, respectively, to define dendritic spines. Spines along dendritic segments were counted and classified starting from 20 to 50 μm from the soma. Spines were classified as immature or mature based on morphology. Long, thin, and filopodia-like spines were classified as immature, whereas mushroom-shaped and stubby spines were classified as mature (Galvez and Greenough, 2005; Majewska et al., 2006; Ron et al., 2011). We cannot rule out the fact that some of the immature spines observed may be in the process of extension or retraction. Spine densities and types were manually counted from at least 10 neurons for each experimental condition, and analysis was performed with the experimenter blinded to the condition.

### Electrophysiology

Whole cell patch-clamp recordings were made on the soma of cortical neurons. For recordings, cells were bathed in artificial cerebrospinal fluid containing (in mM): 140 NaCl, 5 KCl, 2 CaCl_2_, 2 MgCl_2_, 10 HEPES, and 10 glucose (pH 7.4 adjusted with NaOH; 290–310 mOsmol). Recording electrodes (3–5 MΩ) contained a K^+^-based internal solution composed of (in mM): 126 K-gluconate, 4 KCl, 10 HEPES, 4 ATP-Mg, 0.3 GTP-Na_2_, 10 phosphocreatine, and 10 QX-314 bromide (pH 7.2; 280–300 mOsmol). To record miniature excitatory postsynaptic currents (mEPSCs), we blocked action potentials with 1 μM tetrodotoxin (Tocris, R & D Systems; Minneapolis, MN). The membrane potential was held at –70 mV throughout all experiments. Data were amplified and filtered at 2 kHz by a patch-clamp amplifier (Multiclamp 700B), digitalized (DIGIDATA 1440A), stored, and analyzed by pCLAMP (Molecular Devices; Union City, CA). Data were discarded when the input resistance changed >20% during recording.

### Statistical analysis

All statistical analyses were performed using GraphPad Prism software (GraphPad Software, Inc., San Diego, CA). Analysis was performed using Student *t* test for two groups or with ANOVA followed by the appropriate multiple comparisons test for *p*-value adjustment for groups of more than two conditions. Tests used for experiments are clearly stated in figure legends. Statistical significance was set at *p* < 0.05.

### Ethics approval statement

This study was carried out in accordance with the recommendations of the National Institute of Health's Guide for the Care and Use of Laboratory Animals (DHHS Publication No. [NIH] 85-23 and all subsequent revisions thereof) and to the Public Health Service Policy on Humane Care and Use of Laboratory Animals followed by Rutgers Institutional Animal Care and Use Committee. The protocol was approved by the Rutgers Institutional Animal Care and Use Committee.

## Results

### NOS1AP alters actin organization and cell morphology when overexpressed in COS-7 cells

**Figure 2:**
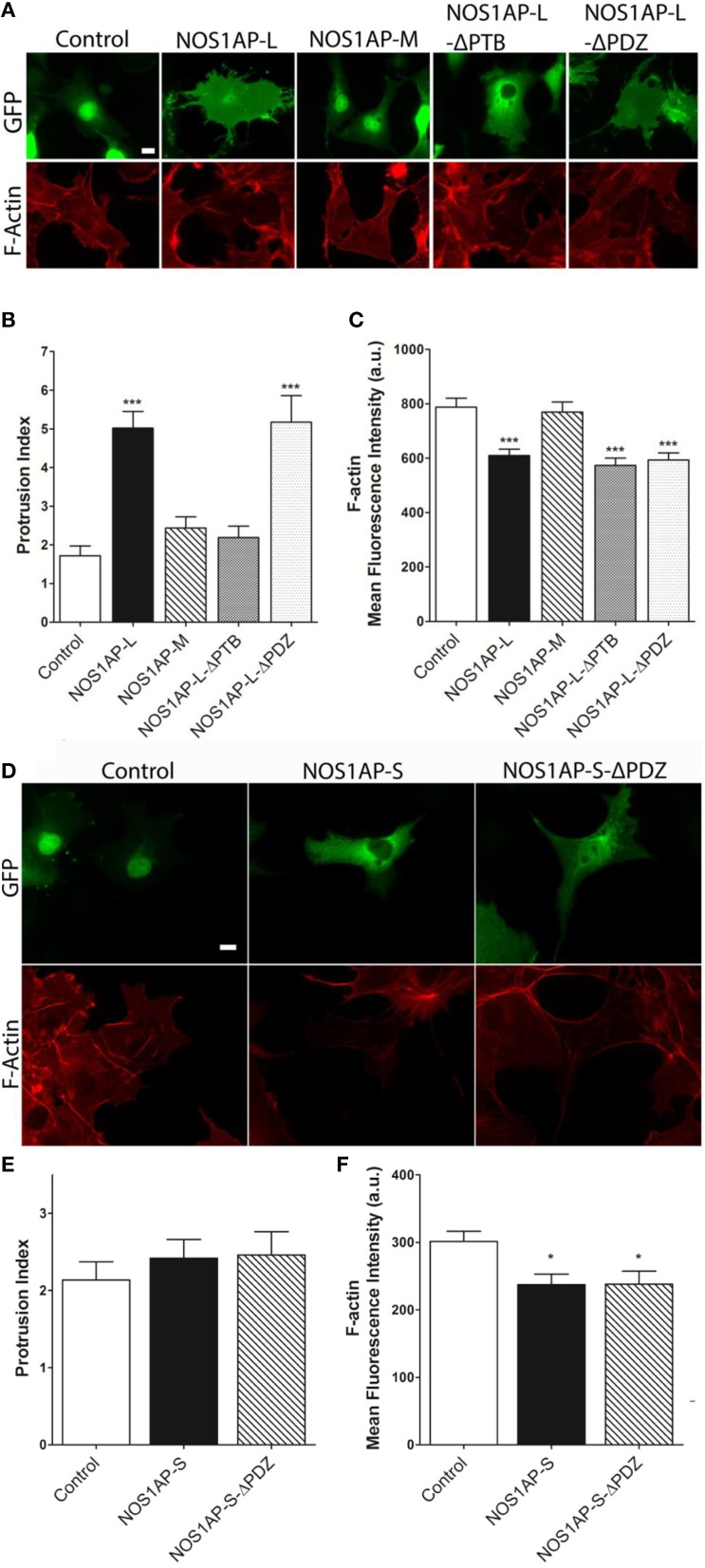
**Effects of NOS1AP-L or NOS1AP-S overexpression on protrusion index and F-actin. (A)** Representative images of Alexa Fluor® 647 phalloidin staining of cells expressing GFP (Control), NOS1AP-L, NOS1AP-M, NOS1AP-L-ΔPTB, or NOS1AP-ΔPDZ. **(B)** Quantitation of protrusion indices from COS-7 cells 48 h after transfection with plasmids encoding the indicated proteins. ^***^p < 0.001 vs. control. *p*-values were determined by one-way ANOVA followed by Dunnett's Multiple Comparisons test. Error bars indicate ± s.e.m from three experiments. *n* = 10 cells, GFP; *n* = 16, NOS1AP-L; *n* = 12, NOS1AP-S; *n* = 10, NOS1AP-M; *n* = 14, NOS1AP-L-ΔPTB; *n* = 11, NOS1AP-ΔPDZ. **(C)** Intracellular F-actin content determined by Alexa Fluor® 647 phalloidin fluorescence intensity 48 h after transfection of COS-7 cells with plasmids encoding the indicated proteins. ^***^p < 0.001 vs. Control. *p*-values were determined by one-way ANOVA followed by Dunnett's Multiple Comparisons test. Error bars indicate ± s.e.m from three experiments. *n* = 36 cells, GFP; *n* = 36, NOS1AP-L; *n* = 36, NOS1AP-S; *n* = 36, NOS1AP-M; *n* = 34, NOS1AP-L-ΔPTB; *n* = 35, NOS1AP-ΔPDZ. Scale bar = 10 μm. **(D)** Representative images of Alexa Fluor® 647 phalloidin staining of cells expressing GFP (Control), NOS1AP-S, or NOS1AP-S-ΔPDZ. **(E)** Quantitation of protrusion indices from COS-7 cells 48 h after transfection with plasmids encoding the indicated proteins. Error bars indicate ± s.e.m from three experiments. *n* = 31 cells, GFP; *n* = 34, NOS1AP-S; *n* = 32, NOS1AP-S-ΔPDZ. **(F)** Intracellular F-actin content determined by Alexa Fluor® 647 phalloidin fluorescence intensity 48 h after transfection of COS-7 cells with plasmids encoding the indicated proteins. ^*^p < 0.05 vs. Control. *p*-values were determined by one-way ANOVA followed by Dunnett's Multiple Comparisons test. Error bars indicate ± s.e.m from three experiments. *n* = 68 cells, GFP; *n* = 49, NOS1AP-S; *n* = 55, NOS1AP-S-ΔPDZ.

We reported that NOS1AP-L and NOS1AP-S regulate dendrite branching (Carrel et al., 2009), and others reported that NOS1AP-L regulates dendritic spine development (Richier et al., 2010) in rat hippocampal neurons. To gain insight into how NOS1AP plays a role in these two cytoskeleton-based processes, we overexpressed NOS1AP-L or NOS1AP-S in COS-7 cells and HEK293T cells and analyzed actin expression 48 h post-transfection. We found no difference in total actin protein when either isoform is overexpressed in either cell line (Figures 1B,C and Supplementary Figure 1). We normalized to GAPDH, which represents total protein, although we find that the total NOS1AP-L expression is lower than GFP and NOS1AP-S expression. During new dendritic branch or spine formation in neurons, distinct types of reorganization of the actin cytoskeleton need to occur (Hotulainen and Hoogenraad, 2010). To investigate the role of NOS1AP isoforms in regulating actin organization, we characterized shape and measured F-actin content of cells overexpressing NOS1AP isoforms. Control cells exhibit typical fibroblast-like morphology (Figure 2A), and the actin cytoskeleton is characterized by the presence of stress fibers and diffuse F-actin immunofluorescence, which we note as “actin organization.” Expression of NOS1AP-L or NOS1AP-L-ΔPDZ, lacking the PDZ-binding motif, induces thin, long, and sometimes branched membrane protrusions (Figures 2A,B), accompanied by a decrease in F-actin content (Figures 2A,C), suggesting that the PDZ-binding motif is non-essential. Cells expressing NOS1AP-S, NOS1AP-S-ΔPDZ, or NOS1AP-L-ΔPTB, lacking the PTB domain, show normal shape, although the organization of actin is altered as shown by the decrease in F-actin staining (Figures 2A,C–F). Expression of NOS1AP-M, the middle region in NOS1AP-L responsible for the effects of NOS1AP-L on dendrite branching (Carrel et al., 2009), has no effect on cell shape or actin organization. Here we show that the PTB domain is responsible for the induction of membrane protrusions observed with NOS1AP-L overexpression, while an unknown shared region between NOS1AP-L and NOS1AP-S is responsible for the reduction in the diffuse F-actin staining. Our data suggest that NOS1AP-L and NOS1AP-S play roles in regulating actin organization via distinct actions.

### NOS1AP-S decreases total Rac1 protein and increases the proportion of inactive cofilin

The Rho family of GTPases, including Rac1, are regulators of dendritic development by influencing the actin cytoskeleton (Nakayama and Luo, 2000; Tashiro et al., 2000; Negishi and Katoh, 2005; Zhang et al., 2005; Sekino et al., 2007). However, reorganization of the actin cytoskeleton may occur in a Rac1-independent manner (Papakonstanti and Stournaras, 2002). It has been reported that NOS1AP-L increases the activation of Rac1 (Richier et al., 2010). To investigate whether NOS1AP-S activates Rac1, we expressed NOS1AP-L or NOS1AP-S in HEK293T cells and measured the levels of GTP-bound Rac1. We did not observe a change in activated Rac1 levels after NOS1AP-S overexpression and failed to observe consistent activation of Rac1 after NOS1AP-L overexpression (data not shown). This may be due to variability in the responsiveness of the cells to Rac1 activation, although cells were subjected to the standard procedure for serum-starvation before examining activation of Rac1.

**Figure 3:**
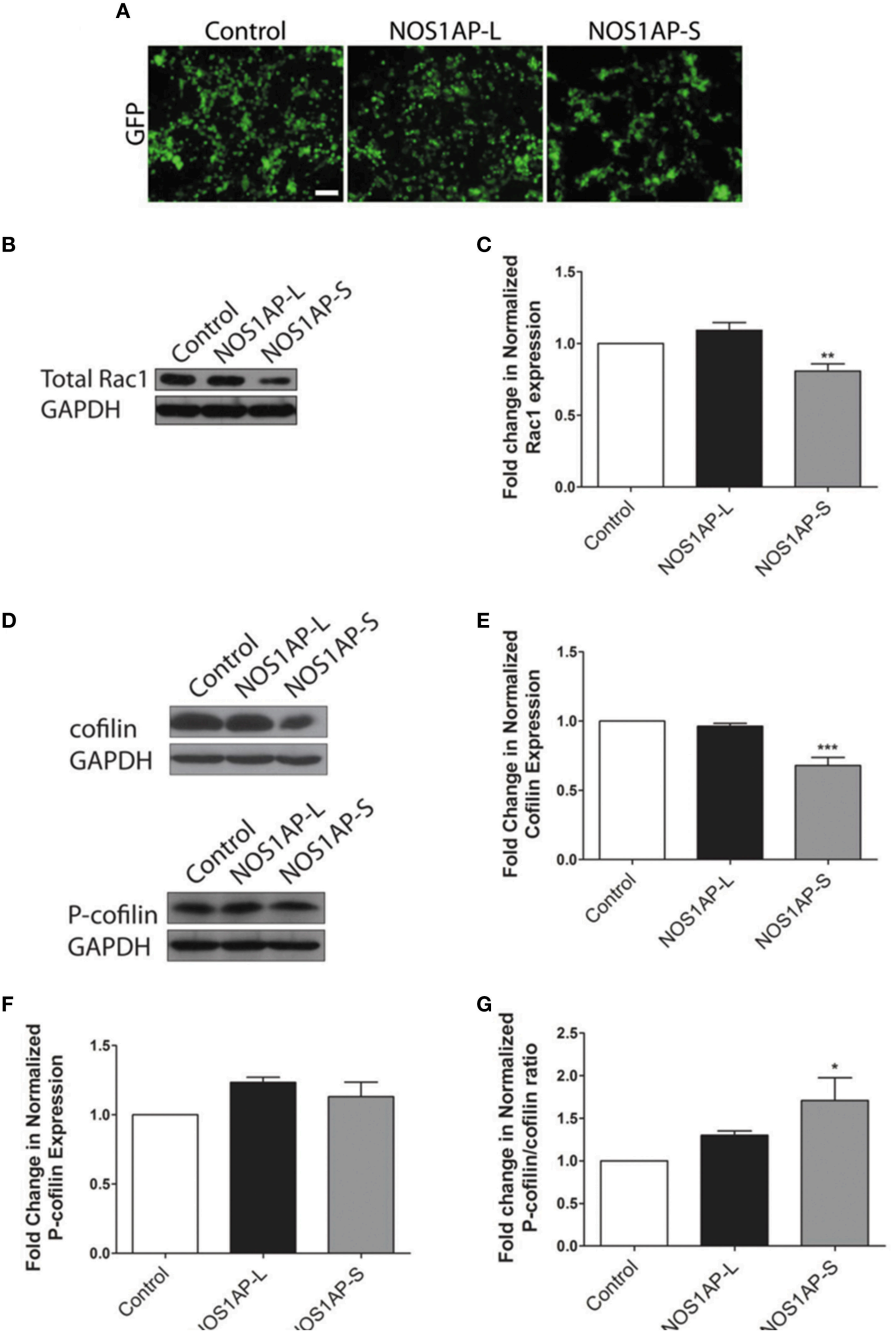
**NOS1AP-S decreases total Rac1 and cofilin protein expression in HEK293T cells. (A)** Representative images of HEK293T cells expressing GFP (control), GFP-NOS1AP-L, or GFP-NOS1AP-S. Scale bar = 100 μm. **(B)** Lysates from cultures of transfected HEK293T cells expressing GFP (control), NOS1AP-L, or NOS1AP-S were resolved by SDS-PAGE and analyzed by Western blotting using antibodies that recognize Rac1 and GAPDH to determine total Rac1 protein levels. Representative blot is shown. **(C)** Relative quantification of Rac1 normalized to control from multiple blots represented in **(B)**. Error bars indicate ± s.e.m. *n* = 9 for all conditions. **(D)** Lysates from cultures of transfected HEK293T cells (different than those in **A)** expressing GFP (control), NOS1AP-L, or NOS1AP-S were resolved by SDS-PAGE and analyzed by Western blotting using antibodies that recognize cofilin, phosphorylated cofilin (P-cofilin), and GAPDH. Representative blots are shown. **(E)** Relative quantification of total cofilin normalized to control from multiple blots represented in **(D)**. Error bars indicate ± s.e.m. *n* = 4 for all conditions. **(F)** Relative quantification of P-cofilin normalized to control from multiple blots represented in **(D)**. Error bars indicate ± s.e.m. *n* = 4 for all conditions. **(G)** Relative quantification of normalized P-cofilin/cofilin ratio from multiple blots represented in **(D)**. Error bars indicate ± s.e.m. *n* = 4 for all conditions. All analyses were performed by first normalizing to GAPDH as an internal loading control and then comparing experimental condition to GFP control condition. ^*^p < 0.05, ^**^p < 0.01, and ^***^p < 0.001 vs. control. *p*-values were determined by one-way ANOVA followed by Dunnett's Multiple Comparisons test.

Activation of Rac1 is not the sole mechanism by which Rac1 may act to alter actin organization. Decreased total Rac1 levels, rather than amount of Rac1 activation, have been shown to inhibit the stabilization of actin-rich protrusions, affecting overall actin organization (Yip et al., 2007). As such, we examined whether overexpression of either NOS1AP isoform results in changes to overall Rac1 levels, with transfection efficiency of cells being similar for all constructs used (Figure 3A). Cells overexpressing NOS1AP-S, but not NOS1AP-L, demonstrate a decrease in total Rac1 protein (Figures 3B,C). To further investigate how a reduction in Rac1 levels, resulting from overexpression of NOS1AP-S, can disrupt actin dynamics, we assessed the activation state of cofilin, a common downstream effector of Rac1 and other Rho family GTPases. Cofilin is a member of the actin depolymerizing factor (ADF)/cofilin family of proteins and enhances the rate of actin filament turnover, both *in vivo* and *in vitro*, by severing, and depolymerizing actin filaments (Carlier et al., 1997; Lappalainen and Drubin, 1997). The activity of cofilin is regulated by phosphorylation of its Ser-3 residue, resulting in its inactivation (Moriyama et al., 1996). When NOS1AP-S is overexpressed, a decrease in total cofilin protein levels results; however, there is no change in levels of the inactive, phosphorylated form of cofilin (P-cofilin; Figures 3D–F). In contrast, overexpression of NOS1AP-L results in no change in total cofilin levels or P-cofilin levels (Figures 3D–F). To elucidate any changes in cofilin activity, we normalized P-cofilin levels to total cofilin, which allows for the analysis of the active, non-phosphorylated form of cofilin. We found that overexpression of NOS1AP-S decreases the levels of the active cofilin, resulting in an increase in the ratio of inactive cofilin to total cofilin (Figure 3G), a measure of cofilin activity standard in the literature. Taken together, our data suggest that NOS1AP-S, but not NOS1AP-L, acts to downregulate levels of total Rac1 and cofilin to promote actin reorganization.

### NOS1AP-L and NOS1AP-S associate with F-actin and regulate actin polymerization

**Figure 4:**
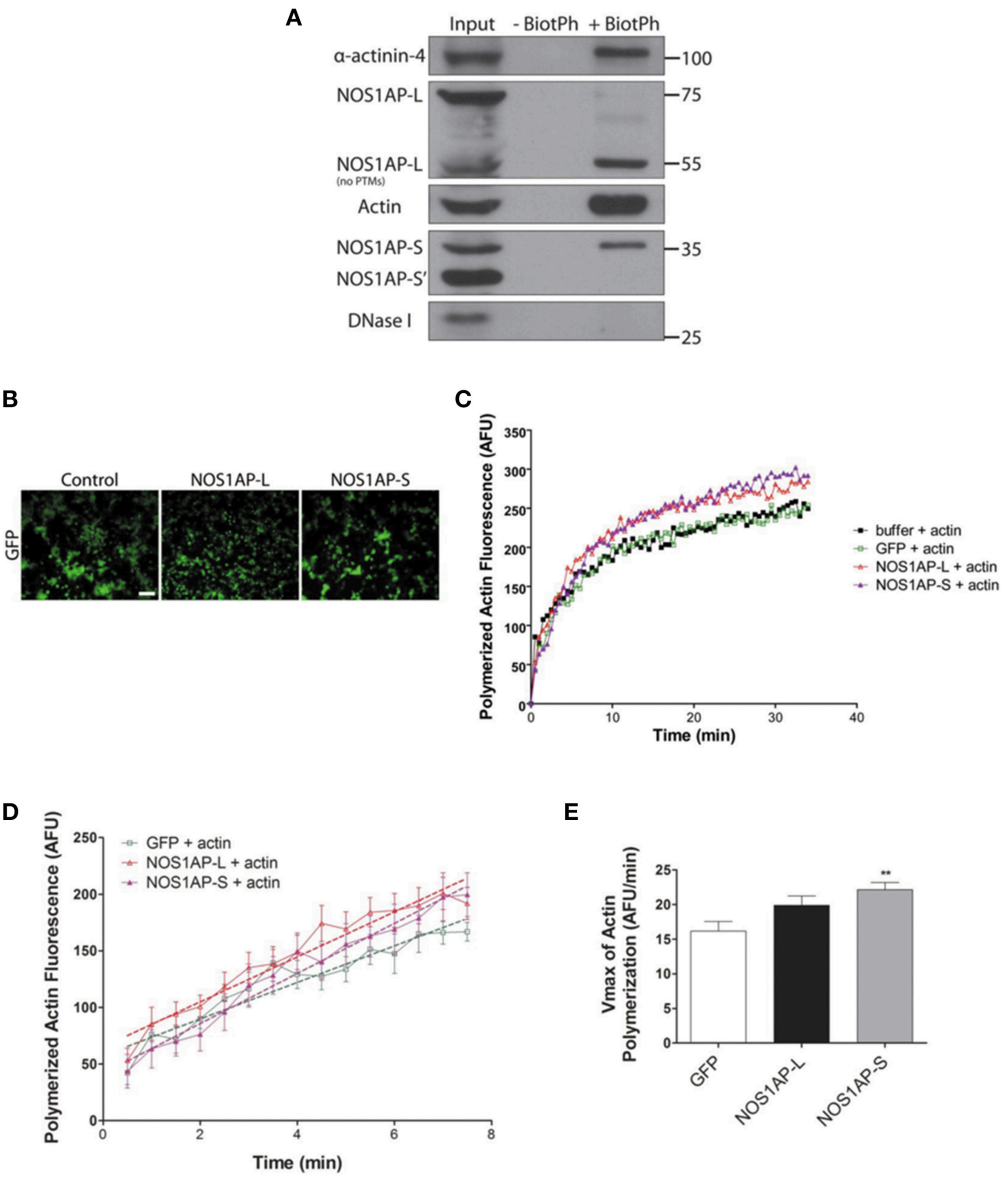
**NOS1AP-L and NOS1AP-S associate with F-actin and promote actin polymerization. (A)** Representative blot showing immunoprecipitation of phalloidin-bound F-actin and NOS1AP from adult rat brain lysate. Tissue was homogenized and cells were lysed in F-actin stabilization buffer. Samples were incubated with or without (negative control) biotinylated-phalloidin followed by precipitation of the captured complexes with streptavidin-linked magnetic beads. Precipitated fractions were then subjected to SDS-PAGE and sequentially immunoblotted to detect NOS1AP isoforms (NOS1AP-L, NOS1AP-S, and NOS1AP-S'), alpha-actinin-4, Dnase I, and actin. Pull down of F-actin also captures alpha-actinin-4 (positive control). BiotPh, biotin–phalloidin; PTMs, post-translational modifications. **(B)** Representative images of HEK293T cells expressing GFP (control), GFP-NOS1AP-L, or GFP-NOS1AP-S. Scale bar = 100 μm. **(C)** Pyrene-actin polymerization assay was performed using a 1:10 mixture of pyrene-labeled muscle actin to unlabeled non-muscle actin. Polymerization of actin was initiated by the addition of polymerization buffer at time 0.5 min. The black trace depicts actin in the presence of lysis buffer alone, green trace depicts effect of adding HEK293T cell lysate from cultures expressing GFP, red trace depicts effect of adding HEK293T cell lysate from cultures expressing GFP-NOS1AP-L, and purple trace depicts effect of adding HEK293T cell lysate from cultures expressing GFP-NOS1AP-S. **(D)** Effects of NOS1AP-L and NOS1AP-S on velocity during the growth phase of actin polymerization. Dotted lines are linear regression of polymerization curves in **(B)** from time 0.5 to 7.5 min to determine V_max_-values. **(E)**, V_max_ data values for actin polymerization kinetics shown in **(D)**. Error bars indicate ± s.e.m. AFU, arbitrary fluorescence units. *n* = 10 polymerization reactions, buffer + actin; *n* = 9, GFP + actin; *n* = 13, NOS1AP-L + actin; *n* = 12, NOS1AP-S + actin. ^**^p < 0.01 vs. GFP + actin. *p*-values were determined by one-way ANOVA followed by Dunnett's Multiple Comparisons test.

Since expression of either NOS1AP isoform can alter F-actin content, we investigated whether NOS1AP-L or NOS1AP-S associates with F-actin in rat brain. Previous studies demonstrated that biotinylated-phalloidin specifically precipitates F-actin (Fulga et al., 2007; Clarke and Mearow, 2013). Tissue extract was incubated with or without (negative control) biotinylated-phalloidin followed by precipitation of captured complexes with streptavidin-linked magnetic beads. Precipitated fractions were subjected to Western blotting to detect F-actin. Pull-down of F-actin (Figure 4A) captured NOS1AP-S and NOS1AP-L lacking post-translational modifications (~55 kDa). Pull-down of F-actin also captured alpha-actinin-4, a known F-actin binding partner (Maruyama and Ebashi, 1965; Drabikowski et al., 1968). DNAse I, which preferentially binds to G-actin (Schafer et al., 1975), and a third isoform of NOS1AP (NOS1AP-S′), previously identified by our laboratory (Hadzimichalis et al., 2010), were not detected in precipitated fractions.

To investigate whether the association of NOS1AP with F-actin influences actin dynamics, as defined by the rate and amount of actin polymerization, we performed *in vitro* actin polymerization assay. Recombinant NOS1AP-L or NOS1AP-S expression in *Escherichia coli* could not be achieved; therefore, HEK293T cell lysates from cultures expressing GFP, NOS1AP-L, or NOS1AP-S were used for actin polymerization assays (Figure 4B). Polymerization of actin was initiated by the addition of polymerization buffer containing 2 mM MgCl and 50 mM KCl at time 0.5 min (Figure 4C). The presence of NOS1AP-L or NOS1AP-S enhances polymerization of F-actin and results in an increased final amount of F-actin (Figure 4C). Using linear regression analysis, the maximum velocity, V_max_ was calculated for the growth phase of actin polymerization (Figures 4D,E). Addition of extracts from cells expressing GFP has no effect on V_max_ using buffer alone as a control (data not shown). Compared to actin polymerization in the presence of lysates from cultures expressing GFP, addition of lysates from cultures expressing NOS1AP-S, but not NOS1AP-L, increases the rate of actin polymerization. Our data suggest that NOS1AP-L and NOS1AP-S regulate actin polymerization, a process necessary for spine formation and maturation, via distinct mechanisms.

### NOS1AP influences spine formation and maturation in rat cortical neurons

**Figure 5:**
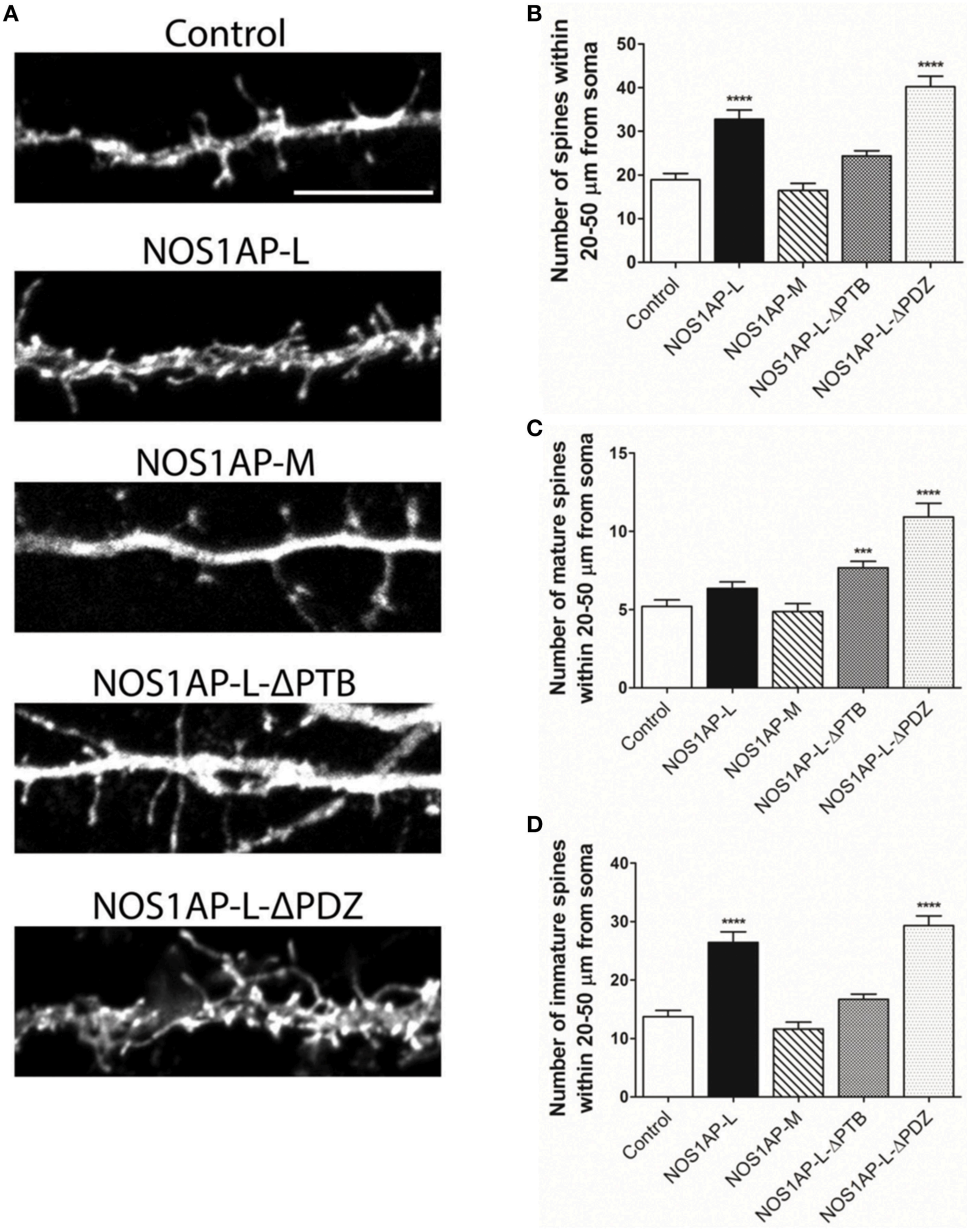
**NOS1AP-L alter spine number and morphology. (A)** Representative images of dendrites from rat cortical neurons (DIV 17) transfected with pCAG-GFP (control), pCAG-GFP-NOS1AP-L, pCAG-GFP-NOS1AP-M, pCAG-GFP-NOS1AP-L-ΔPTB, or pCAG-GFP-NOS1AP-ΔPDZ. Scale bar = 10 μm. **(B)** Number of spines per 30 μm segment in cultured neurons expressing indicated proteins. ^****^p < 0.0001 vs. GFP. *p*-values were determined by one-way ANOVA followed by Bonferroni Multiple Comparisons test. Error bars indicate ± s.e.m. **(C)** Number of mature spines per 30 μm segment in cultured neurons expressing indicated proteins. ^***^p < 0.001 and ^****^p < 0.0001 vs. GFP. *p*-values were determined by one-way ANOVA followed by Bonferroni Multiple Comparisons test. Error bars indicate ± s.e.m. **(D)** Number of immature spines per 30 μm segment in cultured neurons expressing indicated proteins. ^****^p < 0.0001 vs. Control. *p*-values were determined by one-way ANOVA followed by Bonferroni Multiple Comparisons test. Error bars indicate ± s.e.m. *n* = 50 dendrites, GFP; *n* = 41, NOS1AP-L; *n* = 50, NOS1AP-S; *n* = 37, NOS1AP-M; *n* = 55, NOS1AP-L-ΔPTB; *n* = 35, NOS1AP-ΔPDZ. Dendrites were analyzed from 12–20 neurons per condition with 2–3 dendrites/neuron.

**Figure 6:**
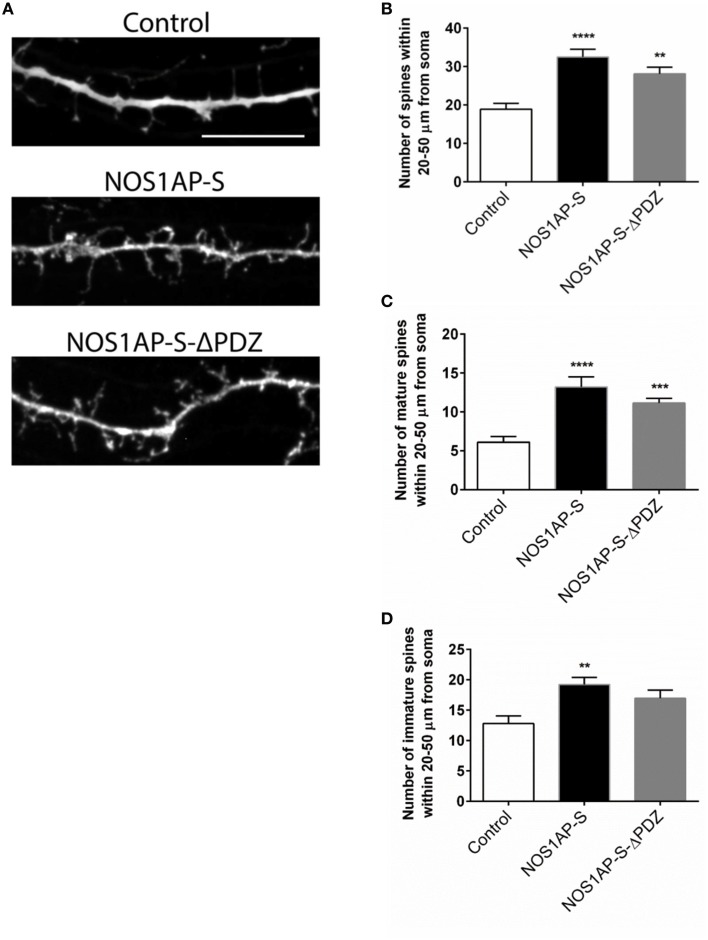
**NOS1AP-S alter spine number and morphology. (A)** Representative images of dendrites from rat cortical neurons (DIV 17) transfected with pCAG-GFP (control), pCAG-GFP-NOS1AP-S, or pCAG-GFP-NOS1AP-S-ΔPDZ. Scale bar = 10 μm. **(B)**, Number of spines per 30 μm segment in cultured neurons expressing indicated proteins. ^**^p < 0.01 and ^****^p < 0.0001 vs. GFP. *p*-values were determined by one-way ANOVA followed by Bonferroni Multiple Comparisons test. Error bars indicate ± s.e.m. **(C)** Number of mature spines per 30 μm segment in cultured neurons expressing indicated proteins. ^***^p < 0.001 and ^****^p < 0.0001 vs. GFP. *p*-values were determined by one-way ANOVA followed by Bonferroni Multiple Comparisons test. Error bars indicate ± s.e.m. **(D)** Number of immature spines per 30 μm segment in cultured neurons expressing indicated proteins. ^**^p < 0.01 vs. Control. *p*-values were determined by one-way ANOVA followed by Bonferroni Multiple Comparisons test. Error bars indicate ± s.e.m. *n* = 30 dendrites, GFP; *n* = 30, NOS1AP-S; *n* = 30, NOS1AP-S-ΔPDZ. Dendrites were analyzed from 10–12 neurons per condition with 2–3 dendrites/neuron.

Our group and others reported that mRNA and protein levels of NOS1AP isoforms are increased in postmortem samples from the DLPFC of subjects with schizophrenia (Xu et al., 2005; Hadzimichalis et al., 2010). We previously reported that expression of NOS1AP-L and NOS1AP-S protein increases during E15 to P14 in rat forebrain, developmental time periods linked to both dendrite branching and spine formation (Carrel et al., 2009). Our data now link NOS1AP-L and NOS1AP-S to actin dynamics, which are important for regulating spine formation and maturation. Thus, we investigated the role of NOS1AP-L and NOS1AP-S in formation and maturation of dendritic spines in cultured rat embryonic cortical neurons. Extensive spine formation and maturation occurs from DIV 14 to DIV 21; therefore, we transfected neurons at DIV 14 and performed spine analysis at DIV 17. Dendritic spines were classified as immature or mature, based on morphology (Galvez and Greenough, 2005). Cortical neurons overexpressing NOS1AP-L display more numerous and thinner dendritic spines, indicative of immature spines, compared to control neurons (Figures 5A,B,D). Expression of NOS1AP-L-ΔPTB eliminated the effects on spine number, suggesting that the PTB domain is responsible for the formation of new, immature spines promoted by NOS1AP-L (Figures 5A,B,D). In comparison, cortical neurons overexpressing NOS1AP-S or NOS1AP-S-ΔPDZ display a greater number of mature and immature spines compared to control neurons (Figures 6A–D). Expression of NOS1AP-L-ΔPTB results in a similar increase in the number of mature spines (Figures 5A,C). Neurons overexpressing NOS1AP-M show no changes to total spine number and spine morphology (Figures 5A–D); however, neurons overexpressing NOS1AP-L-ΔPDZ display increased total spine number, number of immature spines, and number of mature spines, suggesting that the interaction between nNOS and NOS1AP may play a role in regulating spine number and maturation. Taken together, our data suggest that NOS1AP-L and NOS1AP-S have distinct, yet dramatic effects on spine formation and maturation.

### NOS1AP alters synaptic properties in rat cortical neurons

**Figure 7:**
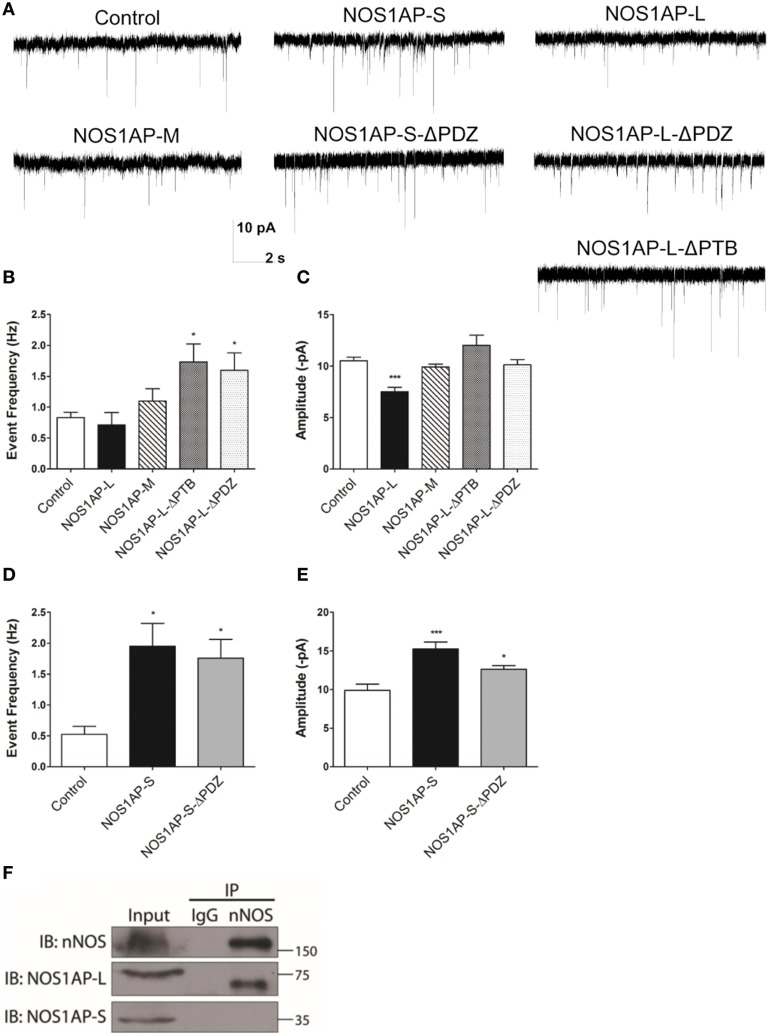
**NOS1AP-S increases synaptic strength, while NOS1AP-L decreases synaptic strength. (A)** Representative tracings of miniature excitatory postsynaptic currents (mEPSCs) from rat cortical neurons (DIV 17) transfected with pCAG-GFP (control), pCAG-GFP-NOS1AP-L, pCAG-GFP-NOS1AP-S, pCAG-GFP-NOS1AP-S-ΔPDZ, pCAG-GFP-NOS1AP-M, pCAG-GFP-NOS1AP-L-ΔPTB, or pCAG-GFP-NOS1AP-ΔPDZ. **(B)** Average frequency of mEPSCs in cultured neurons expressing indicated proteins. **(C)** Average amplitude of mEPSCs in cultured neurons expressing indicated proteins. Error bars indicate s.e.m. *n* = 62 cells, Control; *n* = 14, NOS1AP-L; *n* = 15, NOS1AP-M; *n* = 15, NOS1AP-L-ΔPTB; *n* = 22, NOS1AP-L-ΔPDZ. ^*^p < 0.05; ^***^p < 0.001 vs. Control. *p*-values were determined by one-way ANOVA followed by Bonferroni Multiple Comparisons test. Error bars indicate ± s.e.m. **(D)** Average frequency of mEPSCs in cultured neurons expressing indicated proteins. **(E)** Average amplitude of mEPSCs in cultured neurons expressing indicated proteins. Error bars indicate s.e.m. *n* = 11 cells, Control; *n* = 18, NOS1AP-S; *n* = 19, NOS1AP-S-ΔPDZ. ^*^p < 0.05; ^***^p < 0.001 vs. Control. *p*-values were determined by one-way ANOVA followed by Bonferroni Multiple Comparisons test. Error bars indicate ± s.e.m. **(F)** Immunoprecipitated proteins from rat brain by anti-nNOS or IgG were resolved by SDS-PAGE. Western blots were probed for nNOS and NOS1AP isoforms. A representative Western blot of three individual experiments is shown.

Changes in spine number and morphology promoted by overexpression of NOS1AP-L or NOS1AP-S could lead to distinct functional alterations in synaptic transmission. Thus, we performed whole-cell patch-clamp recordings of miniature excitatory postsynaptic currents (mEPSCs) in cultured rat embryonic cortical neurons. In correspondence with our spine studies, we transfected neurons at DIV 14 and recorded mEPSCs at DIV 17 (Figure 7A). Neurons overexpressing NOS1AP-L show no change in the frequency of mEPSCs but exhibit a significant decrease in the amplitude of mEPSCs (Figures 7B,C). When either NOS1AP-L-ΔPTB or NOS1AP-L-ΔPDZ is overexpressed, the decrease in mEPSC amplitude is lost, suggesting that both the PTB domain and the PDZ-binding motif play roles in altering synaptic properties. Neurons overexpressing NOS1AP-S, NOS1AP-S-ΔPDZ, or NOS1AP-L-ΔPTB demonstrate increased mEPSC frequency with no change in amplitude (Figures 7B–E). Interestingly, overexpression of NOS1AP-L-ΔPDZ also results in an increase in mEPSC frequency, similar to that seen with NOS1AP-S or NOS1AP-S-ΔPDZ overexpression, indicating that the PDZ-binding motif is not responsible for the effect. To determine whether the distinct effects of the two isoforms of NOS1AP are caused by a difference in their interaction with nNOS, we performed co-immunoprecipitation experiments. nNOS and NOS1AP-L co-immunoprecipitate from adult rat brain lysate, whereas NOS1AP-S does not (Figure 7F), demonstrating that only the long isoform of NOS1AP exists in a complex with nNOS in the brain. The observed increase in mEPSC frequency correlates with our findings of increased number of mature spines resulting from NOS1AP-S overexpression. Taken together, our results suggest that NOS1AP-L overexpression decreases synaptic strength and NOS1AP-S overexpression increases synaptic strength and that the effects of the two different isoforms may be due to differences in nNOS binding.

## Discussion

In the present study, we link NOS1AP to the regulation of the actin cytoskeleton, further implicating NOS1AP to the neurodevelopmental hypothesis of schizophrenia (Fatemi and Folsom, 2009; Andreasen, 2010). Improper regulation of the actin cytoskeleton can result in the disruption of several key neurodevelopmental processes. Changes in normal expression of proteins involved in early migration of neurons, axonal and dendritic outgrowth, and synaptogenesis (Fatemi and Folsom, 2009) have been observed in postmortem brain tissues from individuals with schizophrenia, including a study by our group showing that expression of three isoforms of NOS1AP is increased in the DLPFC of individuals with schizophrenia (Hadzimichalis et al., 2010). Two of these isoforms, NOS1AP-L and NOS1AP-S, influence dendrite branching and spine formation (Carrel et al., 2009; Richier et al., 2010). Our results provide mechanistic insight into how NOS1AP can regulate these key neurodevelopmental processes. In addition, we have demonstrated for the first time that NOS1AP can regulate both spine formation and maturation in rat cortical neurons, resulting in changes to synaptic function.

### NOS1AP isoforms and the actin cytoskeleton

**Figure 8:**
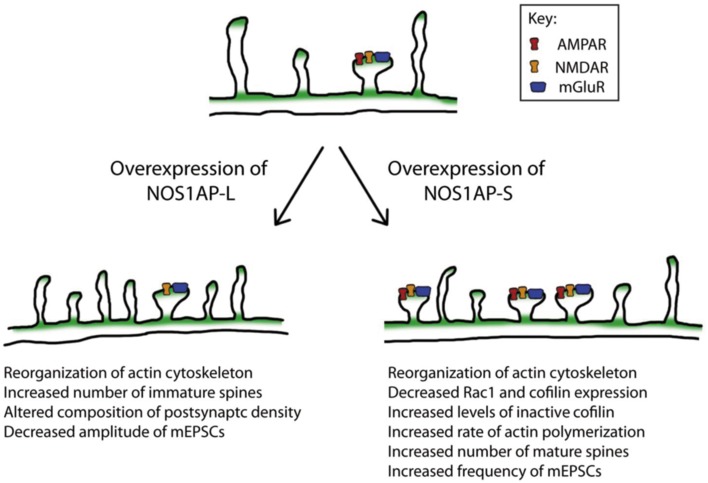
**Model of the actions of NOS1AP-L and NOS1AP-S on the actin cytoskeleton, dendritic spine number and maturity, and synaptic strength**. Overexpression of NOS1AP isoforms result in distinct changes to neurons. A model of action for NOS1AP-L and NOS1AP-S is shown.

Remodeling of the actin cytoskeleton is a common biological pathway shared among several risk factors for schizophrenia (Zhao et al., 2015). Here we report that both NOS1AP-L and NOS1AP-S induce remodeling of the actin cytoskeleton when overexpressed; however, their mechanisms of action are distinct. Both NOS1AP-L and NOS1AP-S associate with F-actin in rat brain, linking both isoforms to the actin cytoskeleton. Specifically, NOS1AP-S, but not NOS1AP-L, exerts its effects on actin by increasing its polymerization rate. Additionally, a previous study reported that NOS1AP-L increases the activation of the small GTPase Rac1 and that the PTB domain of NOS1AP-L is responsible for this activation (Richier et al., 2010). Our results demonstrate that a NOS1AP-L mutant lacking the PTB domain can reorganize the actin cytoskeleton, but cannot induce membrane protrusions as observed with overexpression of NOS1AP-L. These results suggest that the PTB domain of NOS1AP-L is responsible for the induction of membrane protrusions, and that there are multiple regulatory mechanisms by which NOS1AP-L can remodel the actin cytoskeleton. In contrast, we observed a decrease in levels of total Rac1 and cofilin proteins when NOS1AP-S is overexpressed, suggesting that the remodeling of the actin cytoskeleton by NOS1AP-S may be mediated by the regulation of cofilin activity. The increased proportion of inactive cofilin observed with NOS1AP-S overexpression is in agreement with our finding that the rate of actin polymerization is increased in the presence of lysates expressing NOS1AP-S. In summary, we demonstrate that the two NOS1AP isoforms act to regulate the actin cytoskeleton via distinct mechanisms (Figure 8).

Reorganization of the actin cytoskeleton by the Rho family of GTPases is necessary for the remodeling of dendritic spines during development. Both the expression and activity of Rac1 are under tight control in dendrites, and altered levels and activity of Rac1 can influence dendritic development (Urbanska et al., 2008). Indeed, a previous study reported that conditional deletion of Rac1 in mice results in increased thin and stubby spine formation (Golden et al., 2013). Cofilin, a downstream effector of Rac1 and other small GTPases, is important for spine remodeling and synaptic plasticity. Activity-dependent spine growth is coupled to cofilin phosphorylation, which results in actin polymerization (Chen et al., 2007; Calabrese et al., 2014). In addition, both neurons lacking cofilin and neurons with suppressed cofilin activation exhibit a mature spine phenotype (Rust et al., 2010; Pontrello et al., 2012). Here, we show that NOS1AP-S overexpression reduces total Rac1 protein levels. NOS1AP-S overexpression also increases the ratio of phosphorylated cofilin to total cofilin, demonstrating decreased non-phosphorylated cofilin given that total cofilin decreases whereas P-cofilin stays the same. These changes to Rac1 and cofilin can contribute to the observed increase both immature and mature spines in rat cortical neurons. The perturbations in spine morphology resulting from increased NOS1AP isoform expression may have negative consequences for spine development and spine remodeling necessary for synaptic plasticity.

### NOS1AP isoforms and glutamatergic neurotransmission

Numerous studies support the concept that schizophrenia is a disorder of altered connectivity (Narr and Leaver, 2015; Zhou et al., 2015), resulting in the impairment of cognitive, social, and behavioral functions. Connectivity in the brain can be disrupted by the dysregulation of dendritogenesis, spinogenesis, or synaptogenesis. In the present study, we report that overexpression of either NOS1AP-S or NOS1AP-L alters the number and morphology of spines in rat cortical neurons. Specifically, overexpression of NOS1AP-S increases the number of mature spines, which correlates with the down regulation of total Rac1 and cofilin levels. In contrast, overexpression of NOS1AP-L increases the number of immature spines. When the PTB domain of NOS1AP-L is deleted, this effect is lost, further suggesting that the PTB domain is important for the induction of new membrane protrusions. We also found that overexpression of NOS1AP-L-ΔPDZ, which results in a less stable interaction between nNOS and NOS1AP (Jaffrey et al., 1998; Li et al., 2015), results in increased total spine number, number of immature spines, and number of mature spines, suggesting that the interaction between nNOS and NOS1AP may play a role in regulating both spine number and maturation. Additionally, overexpression of NOS1AP-L leads to reduced amplitude of mEPSCs. This change in amplitude may result from a decrease in the amount of transmitter contained in presynaptic vesicles or a change in the function or number of postsynaptic receptors (Turrigiano and Nelson, 2004). Since transfection efficiency is <10% and we do not observe two or more transfected neurons making synaptic contacts with each other, changes in mEPSCs are most likely due to changes in postsynaptic strength. Therefore, the decrease in mEPSC amplitude observed with NOS1AP-L overexpression is suggestive of a reduction in the function or number of postsynaptic glutamate receptors (Turrigiano and Nelson, 2004). This is consistent with our findings that overexpression of NOS1AP-L increases the number of immature spines and can remodel the actin cytoskeleton, a process that regulates the endocytosis of glutamate receptors in spines. Interestingly, overexpression of NOS1AP-L-ΔPDZ does not reduce the amplitude of mEPSCs. Previous studies have shown that activation of the NMDA receptor recruits NOS1AP to nNOS and this interaction enhances NMDA receptor-driven nitrosylation of a nitric oxide effector (Fang et al., 2000; Li et al., 2013). These results suggest that NOS1AP-L may regulate NO signaling through its interaction with NOS1, resulting in the nitrosylation of NMDA receptors, thereby inhibiting the activity of the receptor, leading to reduced amplitude of mEPSCs (Cossenza et al., 2014). Furthermore, we observe an increase in the number of mature spines and an increase in the frequency of mEPSCs with NOS1AP-S or NOS1AP-S-ΔPDZ overexpression, which suggests there is an increase in the number of functional synapses.

### Implications for novel drug development

Individuals with schizophrenia display a variable number of symptoms that fall into three main categories: positive, negative, and cognitive. Currently available antipsychotic medications largely target the dopamine D2 receptor and are most effective in treating the positive symptoms of the illness, while there is little to no improvement in negative or cognitive symptoms (Horacek et al., 2006; Keefe et al., 2007; Davidson et al., 2009; Lally and MacCabe, 2015). While it is important to treat the debilitating effects of the positive symptoms of the illness, it is necessary to find effective treatments for the negative and cognitive symptoms to see long-term improvement in the quality of life for individuals with schizophrenia (Green, 1996; Harvey et al., 1998). As such, investigations into other molecular targets or cellular processes that are altered in these individuals could identify new therapeutic strategies. The DLPFC is a brain region associated with cognitive function and has been implicated in the pathophysiology of schizophrenia, showing perturbations at the anatomical, neuropathological, and neurochemical levels (Bunney and Bunney, 2000). Increased NOS1AP-L and NOS1AP-S protein expression in the DLPFC of individuals with schizophrenia coupled with alterations in neurodevelopmental processes regulated by NOS1AP isoforms suggest that NOS1AP isoforms play a role in cognitive function. To support this notion, it has been reported that healthy individuals carrying a schizophrenia-associated allele within NOS1AP show significantly greater activation of the DLPFC during a task of working memory (Brzustowicz, 2008). Recently, a the creation of a cre recombinase-conditional NOS1AP overexpression transgenic mouse has been reported, but these mice have not been tested for neurocognitive function (Auer et al., 2014). Studying this mouse in the context of neurobehavioral studies would significantly strengthen the link between NOS1AP and cognitive function. Our findings suggest that identifying potential pathways and molecular targets affected by NOS1AP isoforms, as we have done in this study, may prove important for understanding the cognitive deficits observed in schizophrenia and can guide future therapeutic studies.

## Author contributions

KH, PS, MP, and CL were involved in design, execution, interpretation of the data being published, and preparing the article. ND was involved in execution and preparing the article. LB was involved in interpretation of the data being published and preparing the article. BF was involved in design, interpretation of the data being published, and preparing the article.

## Funding

This work was supported by National Institutes of Mental Health grant R01 MH062440 (to LB), a National Alliance for Research on Schizophrenia and Depression 2012 Marion G. Nicholson Distinguished Investigator Award (to BF), and in part, by National Science Foundation grants IBN-0919747 and IBN-1353724 (to BF). KH was supported in part by National Institutes of Health Initiative for Maximizing Student Development Grant 2R25 GM55145, National Institutes of Health Biotechnology Training Grant T32 GM008339-20, and NSF DGE 0801620. PS was supported in part by National Institutes of Health Biotechnology Training Grant T32 GM008339-20. MP was supported in part by a Predoctoral Fellowship from the New Jersey Commission on Brain Injury Research # CBIR15FEL009. CL was supported by a Rutgers University Training Assistant position and an Anne B. and James B. Leathem Summer Fellowship. ND received funding from the Aresty Undergraduate Research Fellowship and Rutgers University School of Arts and Sciences Undergraduate Research Fellowship.

### Conflict of interest statement

Kristina Hernandez, Przemyslaw Swiatkowski, Mihir V. Patel, Chen Liang, and Natasha R. Dudzinski declare that the research was conducted in the absence of any commercial or financial relationships that could be construed as a potential conflict of interest. Dr. Linda M. Brzustowicz serves as a consultant for the Janssen Pharmaceutical Companies of Johnson & Johnson. Drs. Bonnie L. Firestein and Linda M. Brzustowicz reported patent US 12/263,939 titled “Methods and compositions for the diagnosis and treatment of schizophrenia.”

## Supplementary material

Supplementary Figure 1 **Western Blot analysis of GFP expression**. Extracts from cultures of COS-7 cells expressing GFP (control), NOS1AP-L, or NOS1AP-S were resolved by SDS-PAGE and analyzed by Western blotting using an antibody that recognizes GFP. Representative blot is shown. Black asterisks denote GFP fusion proteins. Amersham ECL Rainbow Molecular Weight Markers (GE Healthcare Life Sciences) and PageRuler™ Plus (Thermofisher Scientific) ladders were used.
