# Supplementary figures and images for: Overexpression of Isoforms of Nitric Oxide Synthase 1 Adaptor Protein, Encoded by a Risk Gene for Schizophrenia, Alters Actin Dynamics and Synaptic Function
Source: Front Cell Neurosci. 2016 Feb 2;10:6. doi: 10.3389/fncel.2016.00006 (PMC4735351; doi:10.3389/fncel.2016.00006)

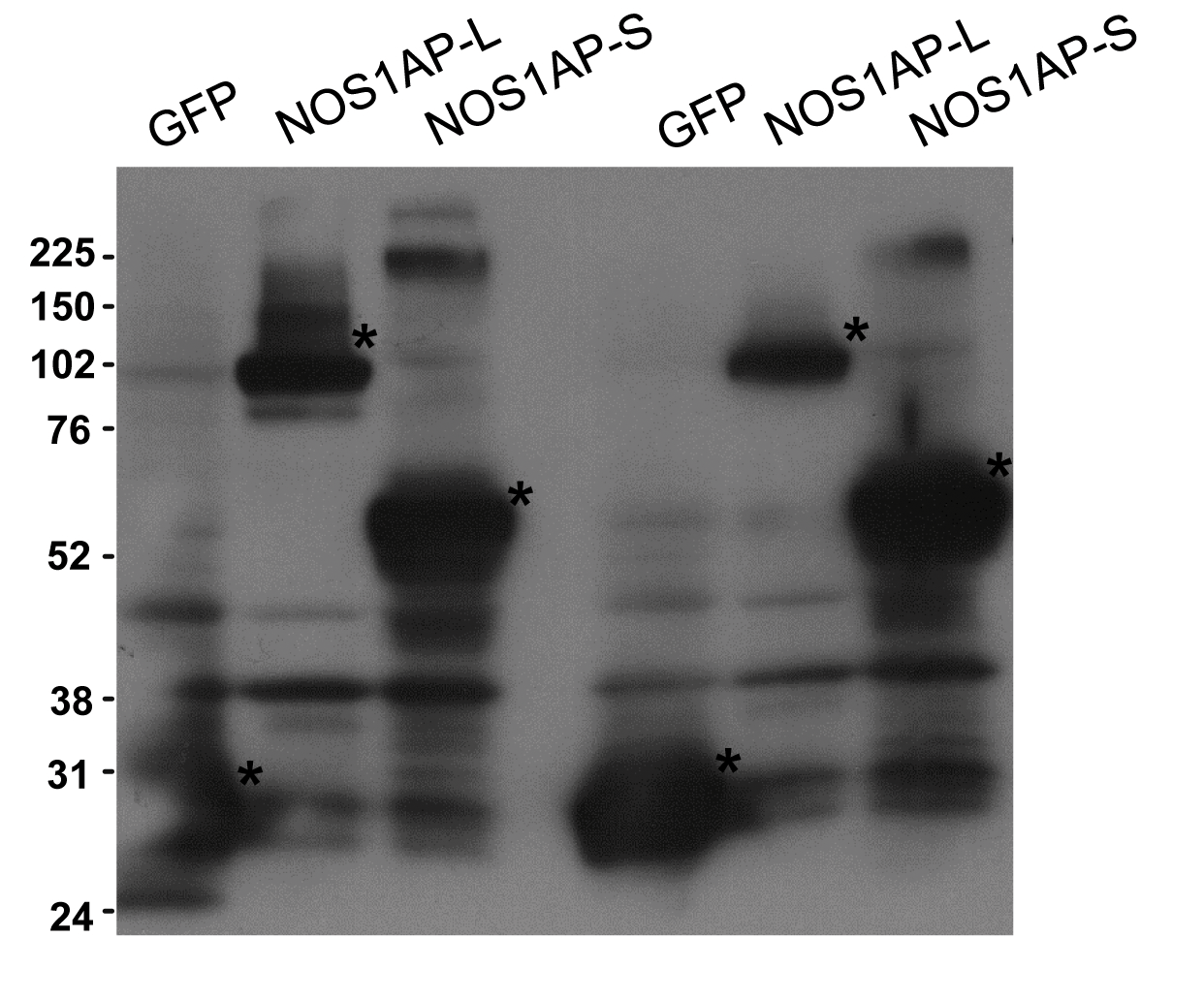

Supplement: Supplementary Figure 1 — Western Blot analysis of GFP expression. Extracts from cultures of COS-7 cells expressing GFP (control), NOS1AP-L, or NOS1AP-S were resolved by SDS-PAGE and analyzed by Western blotting using an antibody that recognizes GFP. Representative blot is shown. Black asterisks denote GFP fusion proteins. Amersham ECL Rainbow Molecular Weight Markers (GE Healthcare Life Sciences) and PageRuler™ Plus (Thermofisher Scientific) ladders were used. [file Image1.TIF]
